# 2078: NGF and TNF-α contribute to oral cancer pain by regulating pro-inflammatory cytokines

**DOI:** 10.1017/cts.2017.197

**Published:** 2018-05-10

**Authors:** Yi Ye, Jihwan Kim, Brian L. Schmidt, Donna G. Albertson, Bradley E. Aouizerat

**Affiliations:** H+H Clinical and Translational Science Institute, New York, NY, USA

## Abstract

OBJECTIVES/SPECIFIC AIMS: We hypothesize that both NGF and TNF-α contribute to oral cancer pain by upregulating pro-nociceptive inflammatory cytokines. METHODS/STUDY POPULATION: In total, 48 oral cancer patients were evaluated and their pain scores were measured using a validated oral cancer pain questionnaire. Presence of perineural invasion (PNI) was identified from patients’ pathology reports. We utilized The NIH Cancer Genome Atlas (TCGA) Head and Neck Cancer cohort to investigate the association between pain and genes related to NGF, TNF-α, and their receptors (TRKA, P75, TNF-α receptor 1, and TNF-α receptor 2) in oral cancer samples by employing PNI as a surrogate for pain. Demographic characteristics, clinical characteristics, and genes were analyzed using logistic regression models. A xenograft cancer pain model was created by inoculating human oral cancer cells (HSC-3) into the mouse hind paw. Mice (n=6 per group) were treated with anti-NGF alone, anti-TNF-α alone, a combination of anti-NGF and anti-TNF-α, or PBS (vehicle control). Nociceptive behaviors were measured using an electronic paw withdrawal assay. Paw volume was measured using a plethysmometer. Cytokines in the paw tissues were measured using a multiplex assay kit with 28 cytokines. RESULTS/ANTICIPATED RESULTS: Oral cancer patients with PNI report significantly more pain compared with patients without PNI in our patient cohort (*p*<0.05). From analysis of TCGA data, we found that PNI is significantly associated with lymphovascular invasion, pathological nodal invasion, and pathological tumor staging (all *p*<0.05). In adjusted models, we observed that the NGF receptor p75NTR (NGFR) and the TNF-α receptor 1 (TNFRSF1A) were associated with PNI (both *p*<0.05) and significantly correlated to each other (*r*=0.25, *p*<0.001). High levels of TNF-α were present in HSC-3 cell lines and the mouse xenograft cancers. In mice with cancer pain, combined treatment with anti-NGF and anti-TNF-α together provided more effective pain control compared with either anti-NGF or anti-TNF-α treatment alone (*p*<0.05). We found significantly increased levels of MIP3a, IL-1b, IL-2, IL-4, IL-28b, IL-23, IL17a, IL-31, and IL-33 in cancer mice compared with normal mice (all *p*<0.05). The combination therapy significantly reduced cytokines MIP3a, IL-1b, IL-4, IL-28b, IL-31, and IL-33 (all *p*<0.05). DISCUSSION/SIGNIFICANCE OF IMPACT: We show that targeting both NGF and TNF-α provides more effective pain relief in an oral cancer model. These results suggest that therapeutic strategies aimed at both pathways could yield improved pain management for oral cancer patients.

